# FIFA World Cup 2022: What can we learn from the inspiring Tokyo 2020 Olympic Games held in COVID-19 times?

**DOI:** 10.5114/biolsport.2022.113293

**Published:** 2022-02-10

**Authors:** Ismail Dergaa, Sarah Musa, Mohamed Romdhani, Amine Souissi, Mariam Ali Abdulmalik, Karim Chamari, Helmi Ben Saad

**Affiliations:** 1 Primary Health Care Corporation (PHCC), Doha, P.O. Box 26555, Qatar; 2 High Institute of Sport and Physical Education of Sfax, Sfax University, Sfax, Tunisia; 3 Physical activity, Sport, and health, UR18JS01, National Observatory of Sports, Tunis, Tunisia; 4 Université de Sousse, Faculté de Médecine de Sousse, Hôpital Farhat HACHED, Laboratoire de Recherche (LR12SP09, Insuffisance Cardiaque), Sousse, Tunisia; 5 Aspetar, Orthopaedic and Sports Medicine Hospital, FIFA Medical Centre of Excellence, Doha P.O. Box 29222, Qatar

**Keywords:** Co-infection, Flurona, Global health, Middle East, Omicron variant, Prevention, Public health, SARS-CoV-2, Soccer, Viral infection

## Abstract

Preparation for the FIFA World Cup 2022 (WC2022) comes in the time of the COVID-19 pandemic. This study aims were to (i) provide a brief overview of the 2020 Tokyo Olympic and Paralympic games in the context of the COVID-19 pandemic, and (ii) highlight the potential challenges and opportunities central to the hosting of the FIFA WC2022. The organisation, public health policies and prevention protocols of the postponed 2020 Olympic/Paralympic Games (Tokyo July-August 2021), the infection rates during the event, as well as the upcoming WC2022 and its related preparations and challenges, were analysed. An unparalleled International Olympic Committee (IOC) effort, together with the Japanese government and people’s perseverance and drive, enabled the safe delivery of the Tokyo Olympic/Paralympic Games, which left a legacy beyond sport. This has been aided by the collection of critical data and lessons learnt throughout the games. The stringent public health policies and especially the tight bubble system for players and their respective delegations have certainly been the key components that ensured the successful containment of COVID-19 within the targeted population. One of the most significant lessons learned from the Tokyo 2020 Olympics is the improvement made in controlling COVID-19 in the context of mass gathering events. Strict infection control strategies to prevent future COVID-19 transmission during the FIFA World Cup 2022 are an immediate priority in Qatar and are constantly being prepared. The planned measures and health care strategies appear to be well adjusted to the risk, especially for the large anticipated number of visitors, and can provide sufficient guarantees to conduct relatively “safe” mega sports events.

## INTRODUCTION

From the start of the pandemic until January 24^th^, 2022, coronavirus disease 2019 (COVID-19) infected over 293 million people worldwide and claimed the lives of more than 5.619 million people [1]. COVID-19 created widespread disruption to global sporting events, many of which have been played behind closed doors, deprived of spectators [2]. In Japan, the 2020 Tokyo Summer Olympics and Paralympics were delayed, to July 2021 [3]. The International Olympic Committee (IOC) made its decision in March 2020, when the number of COVID-19 cases in Japan was 865, compared to 385,000 globally [4, 5]. It was anticipated that mass vaccine deployment would signal the end of the pandemic by 2021; however, Japan and the rest of the world were nowhere near the expected figure of vaccine coverage during the Olympic Games (OG) period [6].

By July 1^st^, 2021, Japan was still in an unstable sanitary situation [6]. The number of cases had surged rapidly to 16,866 [1], and only 14.6% of the total population was fully vaccinated [7]. That was far lower than the estimated 70% needed to hit the herd immunity threshold [8, 9]. The emergence of new COVID-19 variants has made the situation even worse, with their potential to be more transmissible and lethal, especially the Delta variant [8]. However, with all the foreseen challenges, the delayed Tokyo OG and Paralympic Games (PG) were successfully held, ending on Sunday, September 5^th^, 2021 without any reported serious casualties [10]. Apart from limited numbers in some remote locations outside Tokyo and a few thousand schoolchildren in certain of the PG competitions’ facilities, the world has witnessed, for the first time ever, what an Olympics without fans is like [10]. Not surprisingly, Japan had to declare a state of emergency only two weeks ahead of the Tokyo Olympics due to the exponential rise of COVID-19 cases (on the opening ceremony day of the OG, Japan recorded 4200 new infections and by the closing ceremony, that figure had tripled to 12073) [6]. Consequently, Japan retracted and was no longer in a position to host the FIFA Club World Cup 2021 [11]. From an economic perspective, the Tokyo Olympics cost Japan at least $15.4 billion, which is double the initial estimates, making them the most expensive Olympics on record [12].

Despite the continuous decline of newly confirmed cases and deaths globally, the COVID-19 pandemic was still progressing and causing casualties in 2021 [8, 13]. The Tokyo OG has indeed provided a wealth of opportunities for future mega sporting events such as the FIFA World Cup 2022 (WC2022), which will be hosted by Qatar with over 1.7 million expected visitors (fans, staff, players, and media attending the event from November 21^st^ to December 18^th^, 2022) [14].

In this context, many countries worldwide have begun to lift restrictions on large crowds in football stadiums after the OG [15]. Thus, the generated glimmers of hope will hopefully contribute to the transition toward normalcy concerning life in general and sports events in particular. However, genetic changes associated with the emergence of new variants are predicted to affect virus characteristics including transmissibility, disease severity, and immune escape, creating considerable uncertainty about the pandemic destiny [16].

Given the uncertainty surrounding the future of COVID-19 and the countdown to the WC2022, we attempted to present the potential challenges that Qatar may face as it approaches the WC2022, taking the experience of the Tokyo Olympics into account. Thus, the aims of this paper were to *(i)* present a brief overview of the 2020 Tokyo OG/PG in the context of the COVID-19 pandemic, and *(ii)* highlight the potential challenges and opportunities central to the hosting of a safe WC2022 event.

### OG/PG (Tokyo July-August 2021): COVID-19 infection prevention and control protocols

Early detection of a positive case is a critical step in protecting the participants’ health [17]. The COVID-19 countermeasures developed and coordinated by the IOC for Tokyo were a joint initiative between the Partners Task Force, scientists, and organizations across the globe, including the World Health Organization (WHO). During the games, the IOC members and personnel arriving in Japan were almost fully vaccinated or immune. Additionally, 70–80% of the media representatives, as well as 85% of the Olympic Village residents, were vaccinated [18]. Olympic guests, including medical professionals, coaches, and media, needed to have obtained two negative reverse transcription polymerase chain reactions (RT-PCR) within the 96 hours prior to their arrival [18, 19]. Furthermore, each Olympic guest had to undergo a rapid antigen saliva test (RAT) upon arrival and was kept on hold at the airport until the test result was declared negative [18]. However, for those who tested positive, the person was isolated immediately and prohibited from participation until they recovered and a negative COVID-19 RT-PCR result was acknowledged. The length of isolation was dependent on the severity of the presenting symptoms. Furthermore, the IOC has issued sport-specific regulations whenever a positive case is detected during the group stage or in the knockout phase [18]. To help navigate this challenge, no athlete or team would be “disqualified” as a result of a positive COVID-19 test. Instead, they would be marked as “did not start” or a sport-equivalent notation. When possible, the next most qualified athlete or team would substitute for an athlete or team that was unable to compete, allowing the competition to continue as planned. All guests had to install location-based contact tracing apps on their phones and restrict their movements within the country to designated “bubbles” [20]. Their forehead temperatures were taken each time they returned to the Olympic Village (if they were staying there). Athletes sharing lodgings in Tokyo’s Olympic Village, which was hosting roughly 11656 athletes, were subjected to two coronavirus RAT tests a day, and were required to constantly wear face masks, except when sleeping, eating, or competing [20]. Athletes who earned medals had to themselves wear their medals around their necks on the podium, while those who completed competing had to depart the country within two days of their final event [17]. A strict health procedure was also implemented to deal with athletes identified as close acquaintances, which necessitated a massive logistical effort headed by the IOC in close collaboration with the Japanese health institutions [17]. Close contacts were subjected to quarantine and only permitted to leave either for training or competing, employing separate transportation and daily nasopharyngeal RT-PCR testing [20].

### Gap analysis of infection control practices

Tokyo hotels were challenged to keep track of their guests’ activities. It has been reported that hotel operators were subsequently frustrated by their responsibilities in maintaining the bubble around the Olympic delegations [21]. Additionally, the IOC playbooks were deemed imperfect, as many visitors and delegates had not followed the stated guidelines. Failure to track the movements of tens of thousands of visitors, coupled with the likelihood of false-negative results associated with RAT tests as opposed to PCR tests, meant that it was not uncommon to witness a surge of Delta variant transmission in Japan [5]. In a perspective study conducted by Sparrow et al. [5] examining the IOC’s playbooks, the authors concluded that the organization chose cheap methods in place of scientifically validated ones. According to these authors, the IOC’s recommendations were based on an out-of-date understanding of how COVID-19 spreads, namely that the disease is disseminated only by large droplets that fall to the earth swiftly, rather than minute particles that remain and spread in the air [5]. The authors suggested that the IOC and local organizers should take measures to limit aerosol transmission, such as installing hospital-grade air filters or “HEPA filters” in every hotel room, venue, transit vehicle, canteen, and communal space. They recommended that athletes should be accommodated in single rooms and be provided with appropriate facemasks [5]. The authors also reported that face coverings would not protect the athletes as well as those in close-contact environments such as transport vehicles, and further insisted that athletes should use filtering facepiece respirators such as the N95 respirators [5]. In the same alarming paper, the authors mentioned that OG could become a global COVID-19 mega-spreader event [5]. They stated that the IOC and local organizers should do “real-time genetic testing” to ensure that athletes do not unintentionally bring a variation home to unvaccinated, unprotected people with variable or weak healthcare infrastructures [5]. However, and despite all these alarming pre-games statements, and based on data published by the IOC, only three COVID-19 cases have been exported out of Japan (out of the 118,000 who left Japan to get back to their resident countries, i.e., 0.0025%), 14 days after departure from Japan [22, 23]. This has fortunately rendered all the pre-games alarming messages relatively “off-side” with what actually happened in reality with a successful OG/PG event organization with no major health issues reported.

### COVID-19 positive case during the Olympics “within the bubble”

Based on publicly available data, the number of daily reported COVID-19 positive cases during OG/PG was extracted from the OG website (olympics.com, worldometers.info, and ourworldindata.org, last visit: October 15^th^, 2021). A total of 863 positive cases were identified (547 cases at the OG, and 316 at the PG) during the entire event. Out of almost 118,000 Olympic guests, including athletes (n = 11,656) [22], medical professionals, coaches, and media, 547 cases were declared positive during the OG and PG (i.e., 0.46% of positive cases amongst all the Olympic guests).

### Olympics Games

**FIG. 1:**
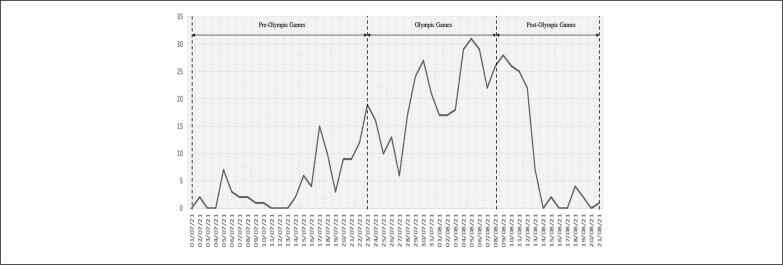
Number of COVID-19 positive cases identified during the Tokyo Olympic Games.

Among the total of 547 positive cases, 88 were identified as pre-OG, 342 during-OG, and 117 post-OG (Figure 1) [22]. The pre-OG (1^st^ to 22^nd^ July) resembled the arrival of participating delegations to Japan. The OG (23^rd^ July to 8^th^ August) corresponded to the inaugurations until the closing ceremony. The post-OG (9^th^ to 21^st^ August) corresponded to the departure of the participating delegations from Japan to their respective countries.

### Paralympic Games

**FIG. 2:**
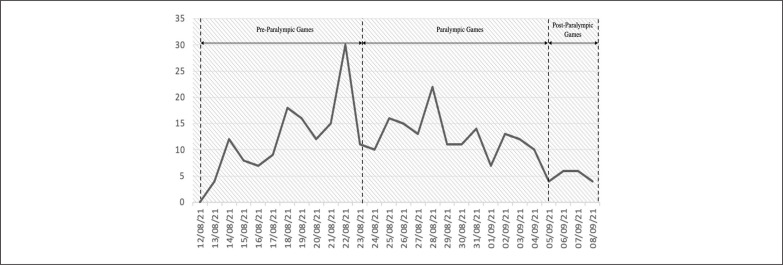
Number of COVID-19 positive cases identified during the Tokyo Paralympic Games.

In comparison to the OG, public awareness and advocacy for the needs of people with disabilities in providing accessible public spaces appeared to have influenced the PG outcomes. Among the total of 316 positive cases, 142 were identified as pre-PG (12^th^ to 23^rd^ August), 158 were during-PG (24^th^ August to 5^th^ September), and 16 were post-PG (6^th^ to 8^th^ September) (Figure 2) [22].

### Positive cases in Japan during the Olympics “outside the bubble”

**FIG. 3:**
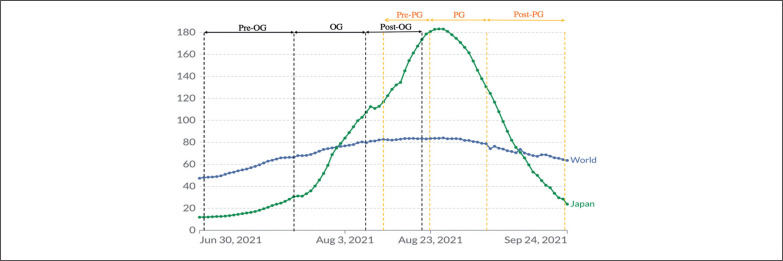
Daily new confirmed COVID-19 cases per million people: comparison between Japan and the world population. OG: Olympic Games. PG: Paralympic games.

In contrast, Japan had around 1500 new cases of COVID-19 on 1^st^ July, 2021; however, a record increase in cases was observed during the OG [1]. The country has witnessed a surge in daily COVID-19 cases and continued to battle a major rise for another two weeks following the Games’ end. The number of daily cases reached a spectacular peak of more than 23,000 by August 24^th^, with the Delta variant being the one to blame [1]. By September 6^th^, 2021, the number of daily cases had dropped to 13,852 and continued to recover six weeks later, with only 326 reported cases by October 22^nd^, 2021 (Figure 3) [22].

### Vaccination

Japan’s vaccination rate against COVID-19 was among the lowest of high income countries by the start of the OG, with only 15% of Japan’s population fully vaccinated, while it reached up to 43% by the end of the event [7]. The coronavirus has overwhelmed Japan’s health care system with thousands of people awaiting for isolation and/or treatment. The fact that COVID-19 vaccines are 90% effective in protecting people means that the risk of infection and transmission in fully vaccinated people cannot be eliminated [8]. An immunization rate of less than 70–80% prior to the OG’s commencement might have contributed to the growing trend in COVID-19 cases during the Olympics [24].

### New strains

### Delta variant

**FIG. 4:**
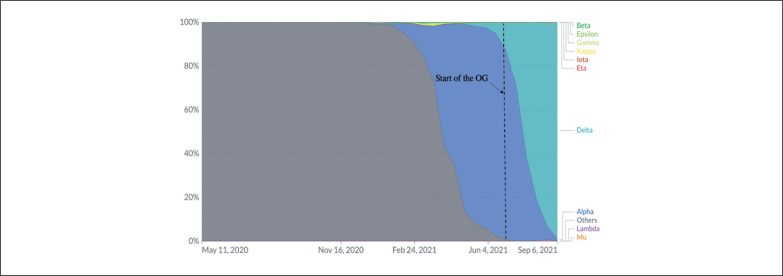
COVID-19 variants in analysed sequence, Japan. OG: Olympic games, Y-axis: percentage of the variant dominance.

The Delta variant (i.e., variant B.1.617.2) has been considered one of the most contagious respiratory viruses ever seen [25, 26]. The rate of virus transmission is estimated by the basic reproduction number (R0) or “R nought”. The influenza virus, for example, has an R0 of roughly two, meaning that each infected individual is estimated to infect two further susceptible contacts [27, 28]. In contrast, the R0 of the original COVID-19 strain was estimated to be somewhere between two and three [27, 29–31]. However, the R0 has risen over the course of the pandemic as the virus evolved and new variants have emerged. However, the estimated R0 of the Delta variant ranges between six and seven, which is three times more contagious than the original COVID-19 strain. According to scientists, the Delta variant is exceedingly difficult to slow down because of its high R0, unless populations reach high levels of immunization. The surge of positive cases in Japan has been linked to the Delta strain (Figure 4) [27]. Indeed, if we take into consideration the evolution of positive cases during the first days of the Pre-OG and the OG, and extrapolate it to the R0 of the Delta strain, the increase of positive cases in Japan could be almost exclusively linked to the Tokyo OG.

### Omicron variant

In November 2021, a new variant coronavirus (variant B.1.1.529) of severe acute respiratory syndrome coronavirus 2 (SARS-CoV-2) emerged and was named Omicron by the WHO [28]. With more than 50 mutations, Omicron has the potential to increase transmissibility, confer resistance to therapeutics, or partially escape infection- or vaccine-induced immunity [29]. Breakthrough infections of Omicron have been reported in many countries, including Botswana, Hong Kong, Israel, Japan, South Korea, the UK, Canada, and the United States (up to January 4^th^ 2022). As a result, several authorities have expanded eligibility for booster doses (e.g., Denmark, England, Finland, France, Germany, Ireland), and/or shortened the minimum interval between completion of a vaccination series and a booster dose (e.g., three months in England, five to six months in Finland, five months in France) [30]. Israel is starting to discuss fourth doses of the vaccine for some populations [31]. Indeed, recent research from South Africa investigating 35,670 suspected reinfections that were identified among 2,796,982 positive RT-PCR swab tests revealed that the Omicron variant was associated with a substantial ability to evade immunity from prior infection [29]. The same researchers raised the question of whether Omicron is also able to evade vaccine-induced immunity and the potential implications of reduced immunity to infection on protection against severe disease and death. On CNBC’s Squawk Box on November 30, 2021, Pfizer Chief Executive Officer Albert Bourla stated that he believes the company’s vaccine will work against the Omicron variant, but that it may provide less protection [32]. Pfizer has begun developing a new vaccine, according to Bourla, in case it is required [32]. The firm has created the first DNA template, which is the first step towards developing a new vaccine. Johnson & Johnson is also putting its Omicron vaccination to the test, according to the company [32]. It is too early to say what effect the Omicron version will have on vaccination protection and previous infection, but Collins of the National Institutes of Health believes Pfizer’s antiviral pill could still function [32]. He stated that there are two methods for determining this: laboratory tests and observation of what occurs in the “real world” [32].

### Uncertainty regarding upcoming variants

Yet, genetic modifications associated with the formation of new variations are likely to affect virus parameters and functionalities such as transmissibility, illness severity, and immune evasion, creating significant ambiguity regarding the pandemic’s fate [16]. If we take the example of the Omicron strain, the virus evolved in a way that it became highly contagious and less severe compared to previous variants [33]. Indeed, the more people are infected by a virus, the higher is the likelihood that the virus will evolve into other strains [34]. With the observed huge number of infected patients, COVID-19 is more likely to acquire more mutations. Even if this is only a speculation, the authors think that if the SARS-CoV-2 undergoes further mutation, COVID-19 could go one of two ways. On one hand, it might mutate into a more lethal, more severe variant, inducing more fatalities, which would create a huge burden for the world and would be more likely to create a huge challenge for the WC2022 to be successfully held. For example, there is a potential risk of COVID-19 and influenza virus co-infection, as described late in December 2021 by an anecdotal media report [35]. On the other hand, COVID-19 could hopefully evolve into other variants, which are less severe, and consequently, the WC2022 would be held safely. As of January 2022, the question “how will the COVID-19 pandemic end?” is still unresolved [36].

### FIFA WC2022: Challenges and opportunities

The number of visitors to Qatar during the WC2022, scheduled to take place in November and December 2022, is projected to reach 1.7 million, exceeding 58.5% of the current present population of Qatar [14]. By hosting a huge tournament amid the COVID-19 pandemic, Qatar is undoubtedly set to face major challenges in the potential wake of new variants’ emergence and global ongoing outbreaks. A stringent infection prevention and control policy has yet to be announced. It is worth mentioning that Qatar effectively held the final of the Amir Cup with over 20,000 fans [2] and 77 matches throughout the Asian Football Cup in December 2020 [37] as well as the FIFA Arab Cup in November and December 2021 [38] with an attendance that exceeded 500,000 in 32 matches. However, with a population of 2.9 million inhabitants, Qatar is confronted to set more credible safety measures. In this context, McLarnon et al. [39] proposed the creation of a COVID-19 passport for professional athletes with details of their previous virus exposure, testing, results, and vaccination. Similarly, Dergaa et al. [8] suggested that a COVID-19 passport should be implemented for spectators as well. As a source of concise information, the COVID-19 passport may serve as a surveillance tool, helping in timely detection and mitigation procedures towards athletes and spectators. A positive COVID-19 case does not merely represent a critical health issue for those players; it also has an impact on their team’s performance and qualification for the next Game(s) to be played [40].

Qatar, one of the Middle East’s smallest countries, is about to make history by hosting the 2022 tournament for the first time in the Arab world and the second time in Asia [14]. According to Worldometer’s analysis of the most recent United Nations data, Qatar’s population is equivalent to 0.04 percent of the global population, with 2.9 million people, the vast majority of whom live in Doha, the capital [14]. The competition has been divided into eight playing locations (Al-Khor, Al-Rayyan, Al-Wakrah, Lusail, Ras Abu Aboud, and three in Doha) spread widely through five out of nine cities in Qatar (Al-Rayyan, Al-Daayen, Al-Wakrah, Dakhira, and Doha) [41]. The stadiums have an average venue capacity of 47,500 seats, and the most distant venues are only 55 kilometres apart [41]. The tournament will feature 32 teams with an expected stadium attendance of over three million people (including Qatari citizens), as with the FIFA WC2018 in Russia and the WC2014 in Brazil [42–44].

Qatar’s health-care spending continues to rise, making it one of the highest healthcare spenders per capita in the Middle East [14]. A mega sporting event can be expected to increase the risk of accidents, injuries, and communicable diseases, including the prevailing COVID-19 infection. Hence, it is vital for the government and hospitals to have mass casualty preparedness plans in place to enhance their emergency response and resiliency. Qatar provides public health services mainly through the Primary Health Care Corporation, which manages 28 health centres and is planning to adopt the WC2022 mass-casualty incident plan in addition to other healthcare institutions that seem to have the potential for mass casualty control and COVID-19 prevention [14, 45]. To ensure that health institutions are well prepared for this mass gathering, any flaws in prior planning are being identified and corrected, and the capacity of Qatar’s hospitals and stockpiles is under enhancement owing to the potential for mass casualties [46]. In addition, some necessary measures are being considered, such as having a sufficient workforce, providing suitable training for medical personnel, and having multilingual services to combat the language barrier [46].

Despite the ambition of reaching herd immunity by the time of the WC2022, COVID-19 continues to pose challenges, especially with the emergence of new variants and breakthrough infections among fully vaccinated individuals. Therefore, alongside the bubble system, the establishment of stringent public health policies remains central to universal source control. The authors believe that the implementation of such stringent measures as imposing a vaccination certificate and/or a recent COVID-19-positive certificate (within six months) and a mandatory COVID-19 immunoglobulin-G antibody titre greater than 33.8 BAU/ml (which are suggestive of protective immunity against COVID-19 [47]) before boarding to Qatar would help the containment of the virus and halt the emergence of further resistant variants globally. Evidence of waning immunity from primary vaccination and/or the vaccine being ineffectual against new variants raises the question of an obligatory recent booster dose as a requirement for entry to Qatar and/or as a match spectator.

In this context, Qatar launched a third-dose vaccine booster campaign in September 2021 to help residents of Qatar live more safely and get prepared for the upcoming WC2022 with less likelihood of serious casualties. For this, it will be very important to aim for sustainable measures through ongoing efforts in COVID-19 surveillance, quarantine, vaccination, and precautionary measures such as wearing masks and social distancing.

## CONCLUSIONS

Japan has witnessed a historical sporting mega event conducted in summer 2021 (Tokyo OG/PG 2020). The experience of Japan and the IOC in dealing with mass gatherings amid COVID-19 offers a rich context for planning future mega sporting events such as the football WC2022. Potential challenges for the upcoming FIFA WC2022 such as the emergence of new variants and breakthrough infections among the vaccinated necessitate more rigorous infection prevention and control measures aiming for early detection, contact tracing and isolation to limit the spread of the virus. Mandatory full vaccination (± booster dose) and/or COVID-19 recovery proof within the last six months and/or immunoglobulin-G antibodies showing protective immunity along with a negative COVID-19 RT-PCR certificate within the last 72 hours prior to departure are suggested upon arrival in Qatar. The authors also recommend imposing rapid antigen testing for fans, staff, players, or media attending the stadium within 24 hours of the match to gain entry to the stadium. A bubble system supplemented by stringent public health policies remains the foremost preventative means concerning the international group of athletes. Taking into account the aforementioned challenges, the planned precautions and health care strategies encountered by Qatar seem to be well adjusted to the risk and may hopefully provide sufficient guarantees to conduct a relatively “safe” FIFA WC2022.
